# Zoonotic disease risk at traditional food markets

**DOI:** 10.1128/jvi.00718-25

**Published:** 2025-07-23

**Authors:** Frida E. Sparaciari, Cadhla Firth, Erik A. Karlsson, Paul F. Horwood

**Affiliations:** 1 Virology Unit, Institut Pasteur du Cambodge 533891, Phnom Penh, Cambodia; 2 College of Science and Engineering, James Cook University 661057, Cairns, Queensland, Australia; 3 CANARIES: Consortium of Animal Networks to Assess Risk of Emerging Infectious Diseases through Enhanced Surveillance; 4 Australian Institute of Tropical Health and Medicine, James Cook University 91846, Cairns, Queensland, Australia; Indiana University Bloomington, Bloomington, Indiana, USA

**Keywords:** traditional food markets, zoonotic diseases, public health, One Health, live animals, food safety, disease surveillance, emerging infectious diseases

## Abstract

Traditional food markets (TFMs) are dynamic and complex systems that play a vital role in societies across the globe. They provide fresh, affordable food, help preserve cultural traditions, and support the livelihoods of millions. However, these markets also present inherent risks associated with the trade of live animals and animal-derived products, including the emergence and spread of zoonotic diseases, which are underreported in these settings. This review explores the dual role of TFMs as essential societal hubs and hotspots for zoonotic diseases, emphasizing the need for surveillance and targeted One Health research on pathogens in these environments. By assessing the health risks associated with the presence of specific animals and their pathogens in TFMs, this review lays the foundation for developing the evidence-based risk assessments and mitigation strategies needed to reduce zoonotic disease risk. Enhancing the safety and sustainability of TFMs through integrated One Health approaches will be crucial for balancing the cultural and economic importance of TFMs with the need for increased global health security.

## INTRODUCTION

Traditional food markets (TFMs) exist in diverse forms and are known by various names, including wet markets, live animal markets (LAMs), and informal markets. Regardless of their designation, they play a vital role in the livelihood of millions of individuals and provide affordable, fresh food to people worldwide. TFMs also serve as critical social and cultural spaces and can even be an attraction for tourists. These markets typically offer a wide range of products, including animal-derived foodstuffs, fresh produce, dried goods, and ready-to-eat meals. In some cases, live animals are housed and slaughtered on-site, contributing to negative perceptions that TFMs pose risks for zoonotic disease emergence (1).

It has been estimated that ~61% of all human pathogens have a zoonotic origin, with zoonoses representing up to 75% of all emerging pathogens over the last decade (2). Zoonotic pathogens can be transmitted to humans through multiple routes, including direct contact with animals, exposure to contaminated urine, feces, or saliva, via aerosols, through ingestion and food from animal origin, and through arthropod vectors such as ticks and mosquitoes. Understanding the etiology, transmission dynamics, and control of zoonotic diseases is crucial for effective prevention and management of these pathogens (3–5). In the past, TFMs have drawn global attention as potential hotspots for emerging pathogens, including severe acute respiratory syndrome coronaviruses (SARS-CoV and SARS-CoV-2), avian influenza viruses (AIVs) (e.g., H5N1), and Nipah virus, as well as the potential future emergence of novel pathogens (often termed “Disease X”) (6, 7). However, TFMs are only one of the drivers behind the heightened perception of zoonotic emerging infectious disease risk, which includes rapid population growth, changes in biodiversity, deforestation, a growing demand for animal protein, wildlife consumption and trade, climate change, and a range of socio-economic and cultural factors. Together, these factors contribute to the risk of zoonotic disease emergence and transmission on a global scale (2, 8).

The World Health Organization (WHO), through its Global Strategy for Food Safety 2022–2030, and the Food and Agriculture Organization of the United Nations (FAO), through its Biosecurity Guide for Live Poultry Markets, have recognized the public health risks associated with TFMs and proposed risk-based interventions such as improving national food control systems, implementing regular cleaning and disinfection protocols, and promoting stakeholder engagement to reduce foodborne and zoonotic disease transmission risks (9–11). In 2021, the Biregional Advocacy Meeting on Risk Mitigation in Traditional Food Markets in the Asia Pacific Region outlined a range of One Health interventions with the goal of increasing food safety and reducing zoonotic disease transmission (12). Interventions are categorized into short-, medium-, and long-term strategies based on the anticipated time between implementation and impact and are designed to be complementary. For instance, improving hygiene and sanitation standards in TFMs is not only critical for reducing the risk of foodborne disease transmission but also for minimizing zoonotic infections from live animals and human-to-human transmission of infectious diseases. The feasibility and timeline for these measures vary by country and depend on governance structures, policy frameworks, and stakeholder coordination. This review aims to examine the role of TFMs in the emergence and transmission of zoonotic diseases, providing a comprehensive synthesis of current evidence. By analyzing the complex interactions between live animal trade, environmental conditions, and pathogen circulation within these markets, this review identifies key risk factors and knowledge gaps, including pathogens that may be present but have not been prioritized in global health discussions. The insights presented here are essential for researchers and public health professionals seeking to advance the understanding of zoonotic dynamics and the broader implications of TFMs in the context of infectious disease emergence.

## OVERVIEW OF TRADITIONAL FOOD MARKETS

**Fig 1:**
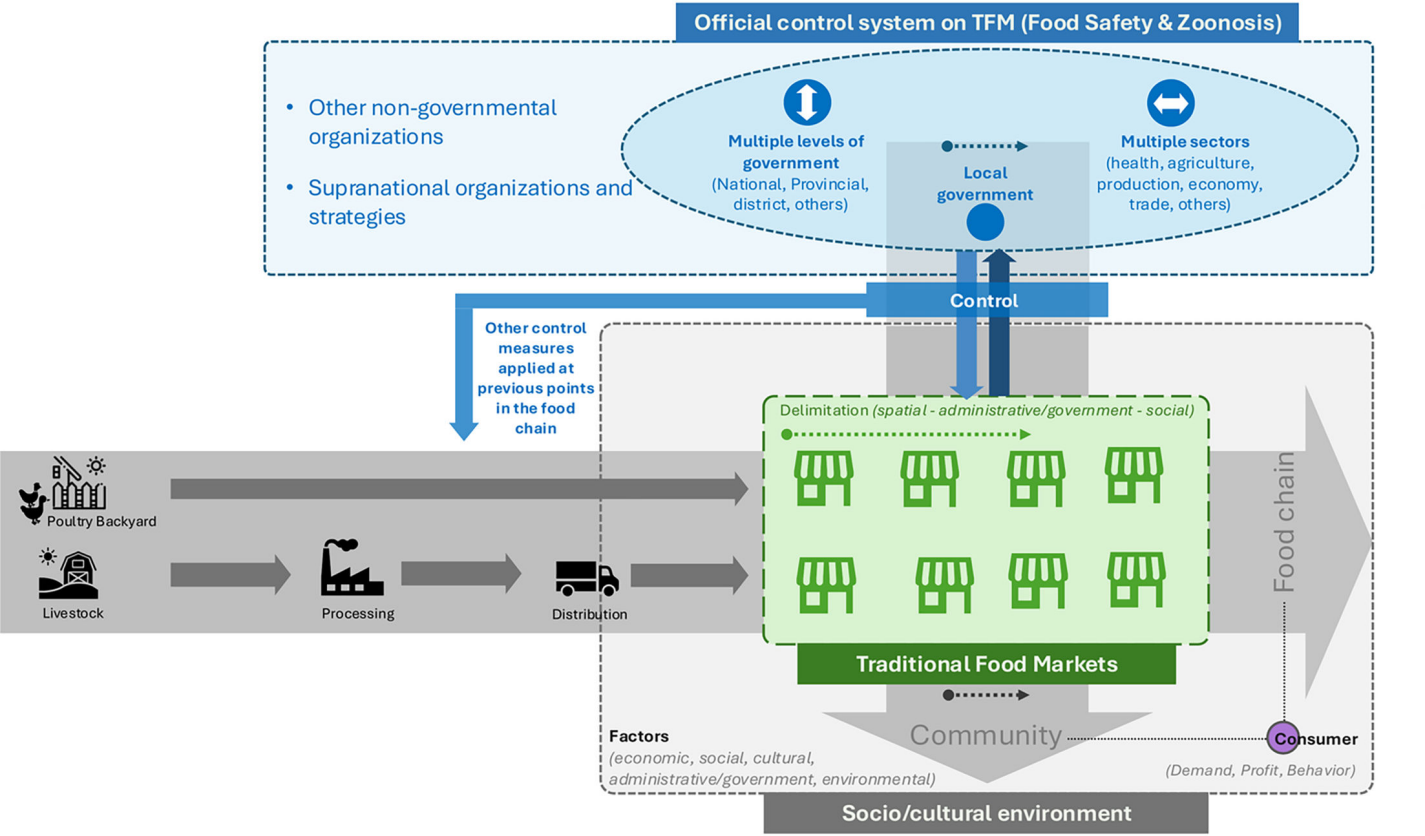
Conceptual framework of a traditional food market system.

TFMs are found worldwide and form an integral part of the broader food supply system, contributing to poverty reduction, food security, and improved nutrition. These markets encompass all aspects of food production and consumption, including economic, health, and environmental impacts (13). In many countries, TFMs operate within informal regulatory structures due to socio-cultural norms, infrastructure, and gaps in policies and laws, leading to inadequate safety inspections and regulatory oversight. As a result, the activities within TFMs may have significant implications for public health and safety (14). To address the complexity intrinsic to the structure and function of TFMs, we developed a conceptual framework of a TFM system and its determinants, which is composed of an official control system, the TFM itself, the food chain, and the social/cultural environment (Fig. 1).

The conceptual framework illustrates the structure and determinants of TFM systems, composed of four interconnected domains: the official control system, the TFM itself, the food chain, and the surrounding socio-cultural environment. The framework suggests that the TFM system is complex and influenced by a variety of factors, including socio-cultural, economic, environmental, and administrative/government elements, at different stages, from production to consumption. Governance operates across various levels and sectors, with national authorities playing key roles, often supported by non-governmental organizations (NGOs) and supranational organizations that provide technical guidance, strategic support, and capacity-building to enhance implementation of control measures.

### The official control system of traditional food markets

With direct links to human, animal, and environmental health, the management of TFMs presents an optimal framework for implementing a One Health strategy, which promotes coordinated, multisectoral actions to address health threats emerging at the human-animal-environment interface (15). The official control systems governing TFMs should be designed to comprehensively manage, monitor, and evaluate emerging public health risks through a multisectoral approach. These control systems incorporate various risk mitigation measures, including legislation, emergency preparedness plans, surveillance systems, communication strategies, laboratory capabilities, inspection protocols, and enforcement mechanisms. These interventions are guided by a collaborative, multisectoral One Health approach that ensures coordination across different levels of government and sectors. Rather than acting as a single measure, this One Health approach serves as a strategic framework that integrates human, animal, and environmental health considerations into the design and implementation of market governance and public health policies (16).

A key challenge in the governance of TFMs is the fragmented regulatory oversight that often results from the involvement of multiple government ministries, each responsible for different aspects of market management. For example, veterinary and food safety inspections in many TFMs fall under separate authorities, leading to limited coordination, overlapping responsibilities, and regulatory gaps—particularly in areas such as live animal handling, on-site slaughter, and waste disposal. These issues are further exacerbated in resource-constrained settings, where vendors operate with minimal external support or access to information on appropriate risk mitigation measures—despite the critical need to balance public health protection with the economic and social roles of TFMs (17). Stall vendors in these small business enterprises often perform multiple essential functions, from animal handling to product sales, yet receive limited coordinated guidance or oversight due to the fragmented nature of official controls across food safety, veterinary, and environmental health sectors. Consequently, they are frequently left to manage complex health risks without adequate training or institutional backing. Furthermore, TFMs often lack direct oversight from a single regulatory authority or specific legislation governing their operations, which contributes to enforcement challenges. In many cases, these markets are either overlooked or not prioritized in regulatory agendas, making the implementation of risk mitigation measures—such as rest days, cleaning and disinfection, use of personal protective equipment, and inspection of food and animals—particularly difficult (18). Nonetheless, some countries, including China, for example, have implemented specific legislation governing TFMs and facilitating the enforcement of mitigation measures. The “1110” policy in China, implemented from 2014 to 2017 to regulate practices in live bird markets (LBMs)—a specific type of TFM with live poultry—was designed to control AIVs. The policy name refers to its key components: “1” daily cleaning, “1” weekly disinfection, “1” monthly market closure, and “0” overnight poultry storage. Surveillance data showed a significant reduction in the prevalence of AIVs in LBMs post-1110 policy, demonstrating the effectiveness of targeted interventions at the market level (19). In Vietnam, the government-led initiative under the Vietnam Avian and Human Influenza Control and Preparedness Project in 2014 aimed to improve biosecurity in LBMs by upgrading market infrastructure and hygiene practices. Implemented in selected LBMs, the intervention included twice-daily disinfection, separation of poultry by source, and structured seller engagement. The intervention markets showed a lower prevalence of AIVs compared to non-intervention markets, and market-level improvements were associated with a reduced risk of virus isolation in birds (OR = 0.76, 95% CI: 0.51–1.14), indicating the potential of structural interventions to support disease control at the market level (20).

The management of TFMs relies on collaboration between local governments, market managers, and vendors to implement risk mitigation measures. Key measures include establishing appropriate infrastructure, applying zoning systems to avoid cross-contamination, ensuring adequate drainage, and proper waste disposal (21). Hygiene practices are essential and should be reinforced through vendor training, regular inspections, and the provision of personal protective equipment and hand sanitizing stations (22). Live animal sales should be conducted in designated areas under veterinary oversight, with strict adherence to animal welfare standards (10). Regulatory compliance and traceability play a critical role in policy enforcement and require strong support from local governments (21). Additionally, vendor and consumer education, transparent communication, and a commitment to continuous improvement are fundamental to ensuring the long-term safety and sustainability of TFMs (23, 24).

### Traditional food markets and the food chain

The food supply chain in TFMs is highly complex and involves a diverse network of suppliers, including small-scale and backyard farmers, middlemen (a person who buys live poultry from the markets and sells to other places), producers of homemade goods, large-scale industries, and markets that may source products from as far away as neighboring countries (25–27). This diversity creates challenges for tracing the origins of products and animals, complicating food safety and regulatory efforts (28). Despite these challenges, TFMs play a crucial role in supporting local economies by providing farmers and producers with direct market access. The agricultural sector plays an important role in reducing poverty and fostering economic development by creating jobs and generating income, particularly for farmers in rural areas (29). TFMs are deeply interconnected within the broader food industry with strong links to the poultry sector. However, few studies have evaluated the supply network or production dynamics of swine and cattle in the context of TFMs, limiting our understanding of their role in these markets (30–33). For example, LBMs in Bangladesh are highly connected, with ~73% of the poultry trading network comprised of LBMs that connect directly with poultry farms and traders, allowing for extensive movement of birds across markets (27). In many regions, markets also rely on informal supply chains. For example, in Vietnam, poultry that are mostly sold in LBMs are commonly raised in independent household and backyard farms (34). Additionally, LBMs serve as important acquisition points where small-scale farmers and households purchase birds for rearing, further strengthening the links between TFMs and local agricultural economies (35). This high level of connectivity within markets makes them particularly vulnerable to disruption during disease outbreaks, such as avian influenza, as pathogens can spread rapidly through these extensive trade networks. For instance, one study in China found that market closures implemented as a control measure during an AIV outbreak resulted in significant losses for the poultry sector, with total costs estimated at ¥7.75 billion (US$1.24 billion). These losses stemmed from disruption of live poultry sales, reduced income from stall rentals, and unrealized sales (36).

### The socio-cultural environment of traditional food markets

The social and cultural environments of TFMs serve as more than just places for the sale of food; they are vibrant hubs for various economic, community, religious, family, and social activities (23, 37). However, their persistence is not only because of tradition but is also driven by continued consumer demand. TFMs provide a unique combination of affordability, freshness, and accessibility that appeals to a broad spectrum of consumers. Many urban populations, particularly in low- and middle-income countries, rely on these markets because they offer fresh food at competitive prices while also supporting local producers (38).

Studies indicate that consumers prioritize TFMs for their ability to see and select fresh products directly, fostering trust in vendors and reinforcing the markets’ role in urban food security. Beyond economic convenience, TFMs are deeply embedded in social structures, where purchasing habits are shaped by cultural preferences, historical practices, and religious customs (39–44).

Alongside their critical role in providing accessible and fresh food, TFMs also provide an avenue for the sale and consumption of wildlife, including products obtained through illegal trade. In Peru, for example, Belén markets function as key distribution points for wild meat, exotic pets, and traditional medicine derived from wildlife (45). Research indicates that wildlife trade in these markets is driven by consumer demand, with local shoppers purchasing illegal wild meat as part of cultural and dietary traditions. The informal nature of TFMs, combined with limited regulatory enforcement, allows the illegal trade of wildlife-origin products to persist, raising concerns about conservation, zoonotic disease risks, and threats to public and animal health (46–48). Despite these challenges, consumer demand for TFMs ensures their continued relevance. Rather than seeking to eliminate these markets, efforts should focus on improving infrastructure, hygiene standards, and monitoring systems to mitigate risks associated with unregulated wildlife trade and to ensure the protection of endangered species, while preserving their economic and cultural significance. While consumers prioritize fresh, affordable, and locally sourced food, TFMs will remain a vital component of global food systems.

## RISK PROFILE ASSOCIATED WITH ZOONOTIC EMERGING DISEASES IN TRADITIONAL FOOD MARKETS

TFMs have faced negative perceptions due to their association with zoonotic disease emergence, including SARS and coronavirus disease 2019 (COVID-19). The profound health and economic impacts of the COVID-19 pandemic highlighted the importance of early detection and ongoing surveillance of zoonotic diseases in higher-risk environments like TFMs (49). Prioritizing the detection and prevention of emerging zoonotic diseases across the food supply chain will be crucial for global health security and the development of effective mitigation measures.

In 2024, the WHO identified 29 pathogens that present the highest level of public health risk based on their potential to cause epidemics and pandemics, of which 16 are zoonotic (50). However, the vast majority of emerging zoonoses are not prioritized by health systems at national or international levels and have, therefore, been labeled as neglected (51). To address this, experts from the US Centers for Disease Control and Prevention (CDC) One Health Office developed the One Health Zoonotic Disease Prioritization process by bringing together representatives from the human, animal, and environmental health sectors to prioritize zoonotic diseases of greatest concern in countries around the world (52). Table 1 describes the transmission routes, animal hosts, and potential for spread for a select subset of zoonotic pathogens relevant to TFMs, as identified by various international organizations and the scientific literature. However, this is not an exhaustive list, nor is it limited to pathogens prioritized by WHO or CDC frameworks. Some pathogens included have not been officially reported as being detected in TFMs but are considered potentially relevant due to vector presence, animal movement, or environmental conditions that may occur in such market settings.

**TABLE 1:** Priority zoonotic pathogens and their relationship with emergence factors and hazards

Pathogen type	Family	Pathogen (50, 53)	WHO/CDC priority	Route of transmission (53–63)	Animal host(s) (57, 64)	Foodborne transmission (53)	Potential for spread in markets
Bacteria	*Leptospiraceae*	*Leptospira* spp.	Yes	Contact with urine (or other body fluids, except saliva) from infected animals. Contact with water, soil, or food contaminated with the urine of infected animals	Rodents and wild and domestic animals (including dogs)	Yes	Could be brought to markets through infected animals, but also risk of scavenging rodents spreading the pathogen in the environment
Bacteria	*Bacillaceae*	*Bacillus anthracis*	Yes	Exposure to infected animals or their food products	Cattle, horses, sheep, pigs, dogs, bison, elk, white-tailed deer, goats, mink	Yes	Exposure to infected animals or their food products
Bacteria	*Brucellaceae*	*Brucella* spp.	Yes	Consumption of unpasteurized milk or milk products or through the inhalation of aerosols and contact with secretions	Cattle, goats, sheep, pigs, and dogs	Yes	Consumption of unpasteurized milk or milk products or through the inhalation of aerosols and contact with secretions
Bacteria	*Enterobacteriaceae*	*Salmonella* spp.	Yes	Ingestion of contaminated food	Domestic animals, birds, dogs	Yes	Spread through contaminated meat, eggs, or other animal products sold in markets
Bacteria	*Campylobacteraceae*	*Campylobacter* spp.	No	Ingestion of undercooked or contaminated food	Cattle, sheep, chickens, turkeys, dogs, cats, mink, ferrets, pigs	Yes	Contaminated poultry or animal products sold in markets can spread infection
Bacteria	*Enterobacteriaceae*	*Escherichia coli*	No	Ingestion of contaminated food or water	Cattle, sheep, pigs, deer, dogs, and poultry	Yes	Contaminated meat or produce in markets can spread the bacteria
Bacteria	*Mycobacteriaceae*	*Mycobacterium bovis, Mycobacterium caprae, Mycobacterium microti*	No	Inhalation or ingestion of contaminated dairy	Cattle, sheep, swine, deer, wild boars, camels, bison	Yes	Unpasteurized dairy or direct contact with infected animals in markets
Bacteria	*Staphylococcaceae*	*Staphylococcus aureus*	No	Direct contact with contaminated surfaces or food	Cattle, birds, pigs, dogs, cats	Yes	Contaminated animal products or surfaces in markets
Bacteria	*Coxiellaceae*	*Coxiella burnetii*	No	Inhalation of aerosols from infected animals	Cattle, sheep, goats, dogs, cats, chickens, wild animals	Yes	Spread through contaminated animal products, wool, or direct contact with infected animals
Bacteria	*Listeriaceae*	*Listeria monocytogenes*	No	Ingestion of contaminated food (dairy, meat)	Cattle, sheep, goats, birds, fish	Yes	Contaminated meat, fish, or dairy in markets
Bacteria	*Francisellaceae*	*Francisella tularensis*	No	Tick or fly bites or direct contact with infected animals	Rabbits, squirrels, muskrats, deer, sheep, bull snakes, wild rodents, beavers, cats, and dogs	Yes	Potential spread through infected game animals or contaminated food/water in markets
Bacteria	*Vibrionaceae*	*Vibrio cholerae*	Yes	Ingestion of contaminated water or seafood	Shellfish (oysters, mussels, and clams), crab, lobster, shrimp, squid, finfish	Yes	Contaminated seafood or water sold in markets can spread cholera
Bacteria	*Enterobacteriaceae*	*Yersinia pestis*	Yes	Flea bites or contact with infected rodents	Rodents (rats, mice, squirrels)	No	Infected rodents or fleas may enter markets and spread the disease
Bacteria	*Enterobacteriaceae*	*Yersinia enterocolitica*	No	Ingestion of contaminated food or water, fecal-oral transmission, and direct contact with infected animals or contaminated environments.	Pigs, rodents, dogs, sheep, oysters, fish, crabs, raw milk	Yes	Contaminated pork products, cross-contamination in markets, and improper refrigeration or hygiene practices.
Bacteria	*Rickettsiaceae*	*Rickettsia prowazekii*	No	Lice bites or contact with contaminated lice feces	Dogs, lambs, goat kids, calves, donkeys, young camels	No	Low risk of market spread, but lice infestations can transmit disease
Bacteria	*Rickettsiaceae*	*Orientia tsutsugamushi*	No	Mite bites (chigger bites)	Rodents	No	Low risk unless infected rodents enter markets, bringing chiggers
Bacteria	*Campylobactereaceae*	*Arcobacter butzleri*	No	Ingestion of contaminated food or water	Poultry, pigs, cattle, seafood	Yes	Contaminated meat, poultry, or seafood in markets may spread infection
Parasitic	*Gnathostomatidae*	*Gnathostoma spinigerum*	No	Ingestion of undercooked fish or meat	Cats, dogs, amphibians, fish, snakes, birds	Yes	Contaminated fish or meat sold in markets could spread the parasite
Parasitic	*Taeniidae*	*Taenia* spp.	No	Ingestion of undercooked meat	Pigs, cattle	Yes	Contaminated meat (pork or beef) containing Taenia larvae
Parasitic	*Trichinellidae*	*Trichinella* spp	No	Ingestion of undercooked meat	Pigs, dogs, cats, rats, and other wild species	Yes	Contaminated meat from infected animals sold in markets can spread the parasite
Parasitic	*Taeniidae*	*Echinococcus granulosus*	No	Ingestion of eggs from contaminated food or waste	Buffaloes, sheep, goats, and adult stray or shepherd dogs	No	Contaminated food is sold or dogs in markets are infected
Parasitic	*Fasciolidae*	*Fasciola hepatica*	No	Ingestion of contaminated water or plants	Cattle, sheep, goats, and other ruminants	Yes	Contaminated vegetables or water sold in markets can spread the parasite
Parasitic	*Strongyloididae*	*Strongyloides* spp.	No	Skin penetration by larvae in contaminated soil or water	Primates, dogs, cats, some farm animals	No	Contaminated soil or animals enter markets
Protozoa	*Hexamitidae*	*Giardia* spp.	No	Ingestion of contaminated water or plants	Dogs, cats, ruminants, pigs	Yes	Contaminated water or food sold in markets can spread the parasite
Protozoa	*Cryptosporidiidae*	*Cryptosporidium* spp.	No	Ingestion of contaminated water or food	Cattle, sheep, pigs, goats, horses, deer	Yes	Contaminated produce or water in markets may spread the parasite
Protozoa	*Sarcocystidae*	*Toxoplasma gondii*	No	Ingestion of undercooked meat or contaminated food	Pigs, sheep, goats, poultry, rabbits	Yes	Infected meat sold in markets could spread toxoplasmosis
Protozoa	*Trypanosomatidae*	*Leishmania* spp.	No	Sandfly bites	Cats, dogs, horses, and bats	No	Infected animals or sandflies are present in markets
Protozoa	*Trypanosomatidae*	*Trypanosoma cruzi*	No	Kissing bug bites or ingestion of contaminated food	Domestic pigs and cats, wildlife reservoirs include opossums, armadillos, raccoons, woodrats	Potential	Potential spread through contamination of food or via vectors in markets
Virus	*Nairoviridae*	Crimean-Congo hemorrhagic fever virus	Yes	Tick bites or exposure to blood or tissues of viremic patients or livestock	Wild and domestic animals, such as cattle, goats, sheep, hares	No	Live ruminants brought to markets could spread virus through body fluids or vectors
Virus	*Filoviridae*	Ebola virus and Marburg virus	Yes	Contact with bodily fluids of infected animals	Non-human primates, bats	No	Sale of live exotic animals or bushmeat brings the pathogen close to humans
Virus	*Arenaviridae*	Lassa fever virus	Yes	Exposure to urine or feces of infected rats	Rodents	Yes	Food products that have been contaminated with the excreta of the infected rodents
Virus	*Coronaviridae*	Zoonotic coronaviruses	Yes	Intermediate hosts	Bats, other mammals	Potential	Large variety of coronaviruses could be brought by live animals taken to the market; some of these viruses may have zoonotic potential
Virus	*Paramyxoviridae*	*Henipaviruses*	Yes	Contact with urine in bats, from infected animals (horses, pigs, and humans) or contaminated foods, human-to-human (Nipah)	Bats, pigs (Nipah), horses (Hendra)	Yes	Contaminated food products or live animals sold
Virus	*Phenuiviridae*	Rift Valley fever virus	Yes	Bites from infected mosquitoes or contact with the blood or organs of infected animals	Buffaloes, camels, cattle, goats, sheep	No	Wet markets could provide breeding grounds for mosquitoes in urban settings
Virus	*Orthomyxoviridae*	Zoonotic influenza viruses	Yes	Contact with infected animals or contaminated environments	Ducks, chickens, wild birds, pigs	Potential	Infected animals can transmit the virus to humans
Virus	*Rhabdoviridae*	Bat lyssaviruses and rabies virus	Yes	Animal bites or blood infection while butchering animals	Dogs and carnivores, bats, other infected mammals	No	Risk from bites from live carnivores or bats in markets. Similarly, markets may attract scavenging dogs, increasing risks for bites
Virus	*Hantaviridae*	*Hantaviruses*	Yes	Contact through the inhalation of aerosols and contact with secretions (49)	Rodents, shrews, moles, bats	Yes	Reservoir animals may be sold at markets, scavenging rodents may also bring the pathogens close to the markets and contaminate products
Virus	*Picornaviridae*	Hepatitis E virus	Yes	Contact with infected animals and transmitted by the fecal-oral route (50)	Domestic pigs, wild boar, and maybe other animal species	Yes	Spread through food products or contacts with live animals at market
Virus	*Flaviviridae*	Zika virus	Yes	Bites from infected mosquitoes	Apes, monkeys	No	Wet markets could provide breeding grounds for mosquitoes in urban settings
Virus	*Flaviviridae*	Dengue virus	Yes	Bites from infected mosquitoes	Fruit bats, monkeys	No	Wet markets could provide breeding grounds for mosquitoes in urban settings
Virus	*Poxviridae*	*mpox*	Yes	Direct contact with infected animals	Squirrels, Gambian poached rats, dormice, different species of monkeys	No	Wet market sells live animals, including rodents and other wildlife, which are known carriers or potential reservoirs
Virus	*Togaviridae*	Chikungunya virus	Yes	Bites from infected mosquitoes	Monkeys, birds, rodents	No	Wet markets could provide breeding grounds for mosquitoes in urban settings
Virus	*Flaviviridae*	Japanese encephalitis virus	Yes	Bites from infected mosquitoes	Pigs, birds	No	Wet markets could provide breeding grounds for mosquitoes in urban settings
Virus	*Flaviviridae*	West Nile virus	Yes	Bites from infected mosquitoes	Birds (primary reservoir), horses	No	Wet markets could provide breeding grounds for mosquitoes in urban settings
Virus	*Flaviviridae*	Yellow fever virus	Yes	Bites from infected mosquitoes	Primates (both humans and non-human primates)	No	Wet markets could provide breeding grounds for mosquitoes in urban settings
Virus	*Flaviviridae*	*Orthoflavivirus kyasanurense*	Yes	Tick bites	Monkeys, rodents, cattle, goats	No	Wet markets could provide breeding grounds for mosquitoes in urban settings

## ZOONOTIC PATHOGEN THREATS IN TRADITIONAL FOOD MARKETS

Live animal markets (LAMs) (65) are a type of TFM containing conditions that may support the emergence and spread of zoonotic disease if not managed appropriately, especially when animals are housed in poor conditions, subjected to stress, and mixed with other species without proper management (66). The common practice of stacking animal cages in close proximity may also increase the likelihood of pathogen transmission between animals and species. Additionally, close contact between live animals, market vendors, and consumers, many of whom purchase animals for home slaughter or traditional medicine, further heightens the risk of zoonotic transmission associated with TFMs. Traditional medicinal practices frequently involve the use of raw or minimally processed animal parts, which can serve as additional routes of exposure to zoonotic pathogens, particularly in the absence of hygiene controls (67). A large diversity of pathogens, including viruses, bacteria, vector-borne pathogens, and parasites, has been reported in animals, humans, and LAM environments (68).

In these environments, live animals are often kept in cages or pens and are slaughtered and dressed in open market areas, resulting in these areas becoming contaminated with animal fluids, feces, and other waste (69). Often, LAMs also contain ready-to-eat food, and many pathogens can cause foodborne illnesses through infected or contaminated food products (e.g., meat or meat products, eggs, dairy products) during processing, or through insufficient hygiene practices (70). In some parts of Asia and Africa, LAMs may also sell wildlife, both alive and as derived products, much of which is sourced from illegal and/or unregulated trade, including species like turtles, birds, pangolins, civets, primates, and weasels (71). Illegal wildlife trade has been implicated in the transmission of diseases among both animals and humans frequenting these markets (69, 72).

### Viral threats in traditional food markets

LAMs contain conditions that have been associated with an increased risk of virus transmission, amplification where the virus multiplies more within hosts, making it easier to spread to new hosts (68). The frequent and close interactions between humans and animals in these markets, whether through occupational exposure, local trade, or tourism, may further facilitate the spillover of viral pathogens. This dynamic not only increases the risk of initial cross-species transmission but also enhances the potential for viruses to spread rapidly within and beyond the market environment, posing significant public health threats.

#### Avian influenza viruses

AIVs remain a significant global health concern, as demonstrated by the ongoing global panzootic caused by highly pathogenic avian influenza virus H5N1, clade 2.3.4.4b. Over the past decades, recurrent waves of infection have impacted domestic and wild bird populations across the world, as well as several mammalian species, posing serious risks to biodiversity and human health (73). AIVs possess a genome consisting of eight distinct gene segments, which can undergo genetic reassortment when different AIV subtypes co-infect the same cell. Through reassortment, new AIVs can be generated that may display novel phenotypes, including increased potential for cross-species transmission. Reassortment is a major driver of the rapid evolution observed in AIVs and facilitates the emergence of new subtypes with epidemic and zoonotic potential. Both reassortant and non-reassortant strains may also acquire point mutations that enhance immune evasion, cellular tropism, or other mechanisms of host adaptation, increasing the risk of cross-species transmission and the potential for outbreaks in both human and animal populations (74–77).

Numerous studies have reported high prevalences of AIVs in LBM settings. In Bangladesh, multiple studies indicate an overall AIV prevalence of 31%–49% in various LBMs, with species mixing and poor waste management contributing to widespread environmental contamination (17, 78, 79). In Egypt, 28% of poultry samples from LBMs tested positive, predominantly for H5N1 (80), whereas in Vietnam, just 14% of poultry samples were positive for AIVs, including subtypes H5N1 and H9N2 (81). These findings highlight the complex interactions between environmental conditions, species diversity, and trade practices that drive AIV persistence and evolution in LBMs.

The image shows carcass wash water processing in the poultry slaughter areas, where discarded feathers and other waste materials accumulate. These high-risk zones exemplify the potential for environmental contamination and human exposure to AIVs, as supported by high detection rates in carcass wash water and other environmental surface samples.

**Fig 2:**
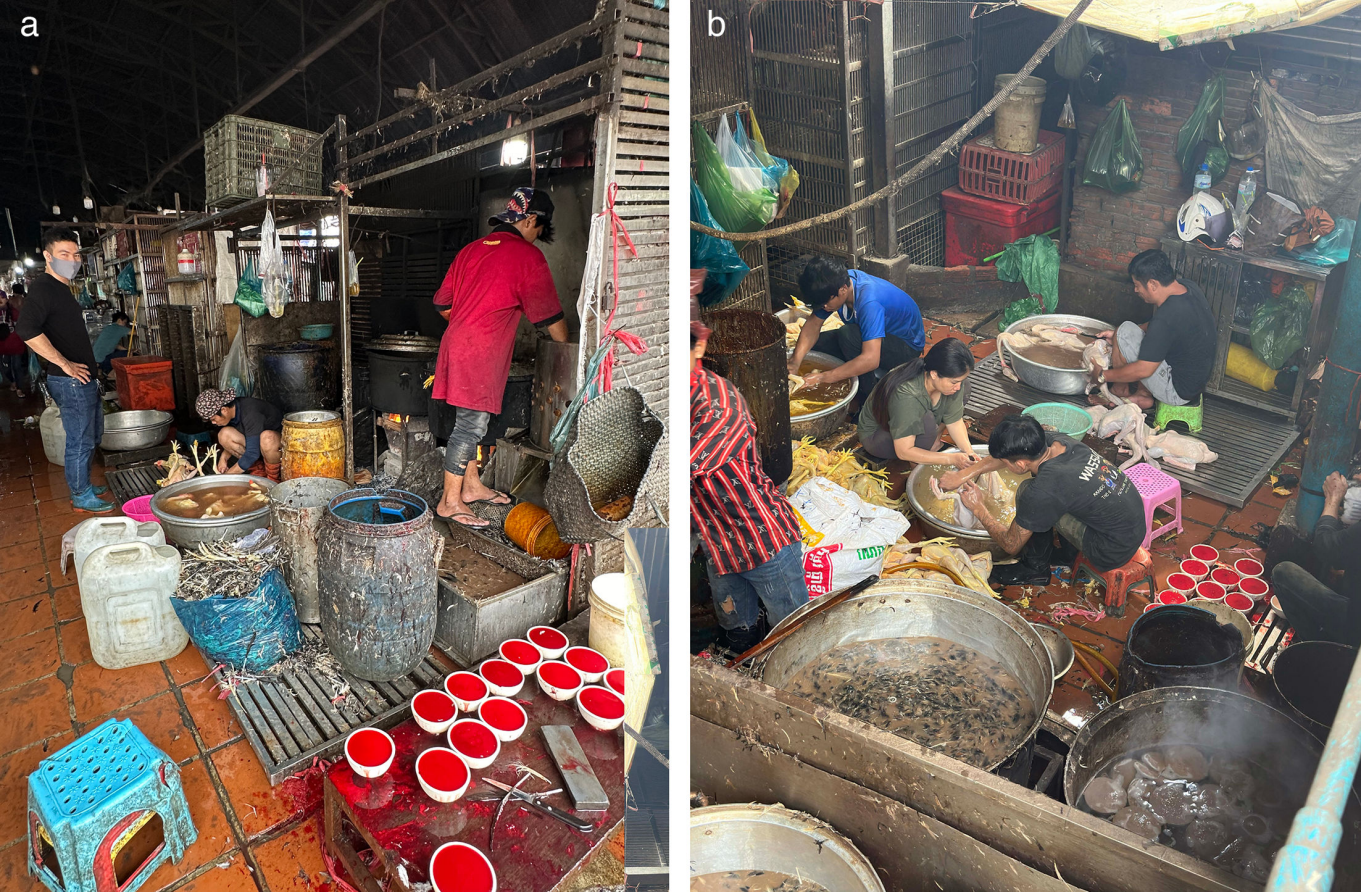
Carcass wash water processing in the poultry slaughter areas. (a) A slaughter area in an LBM. (b) LBM workers manually defeathering birds. Photo courtesy of Frida E. Sparaciari from Virology Unit, Institut Pasteur du Cambodge.

A study in Cambodia used environmental sampling in LBMs to reveal high levels of environmental contamination with AIVs (77). The highest AIV detection rates were observed in carcass wash water samples (Fig. 2), which are pooled samples from multiple slaughtered birds. Poultry drinking water samples were also identified as valuable for AIV surveillance, as 50% of samples were positive and H5N1 was detected more frequently than in carcass wash water. High rates of AIV detection were also found in soil/mud and discarded feather samples. These findings indicate a substantial risk of human exposure to AIVs in LBMs, with carcass wash water and poultry drinking water potentially the most effective samples for monitoring (78, 82).

#### Coronaviruses

LAMs have been identified as significant sources of zoonotic coronaviruses associated with outbreaks in people, particularly Middle East respiratory syndrome coronavirus (MERS-CoV), SARS-CoV, and SARS-CoV-2. Coronaviruses are positive-sense, single-stranded RNA viruses that consist of multiple genera, including *Alphacoronavirus*, *Betacoronavirus*, *Gammacoronavirus,* and *Deltacoronavirus*. Both alphacoronaviruses and betacoronaviruses can cause disease in humans and animals. Bats are recognized as important reservoir hosts that harbor a diversity of coronaviruses and have long-standing associations. To date, more than 4,800 coronavirus sequences have been detected in bats, distributed worldwide (83).

The outbreak of SARS in 2002–2003 was one of the first major disease events that raised concerns about the role of LAMs in the spread of zoonotic diseases. The etiologic agent behind this epidemic, SARS-CoV, is thought to have originated in bats and likely jumped to humans working in LAMs through an intermediate host, putatively identified at the time as the palm civet (*Paguma larvata*), sold in LAMs in southern China (84, 85).

Environmental surveillance (ES) at the Huanan Seafood Market in Wuhan, China, revealed widespread contamination of SARS-CoV-2 across surfaces during the early stages of the outbreak. Between January and March 2020, a total of 923 environmental samples were collected, with 8% testing positive for SARS-CoV-2. Notably, 87.5% of positive samples were concentrated in the market’s western zone, where live animals including birds, reptiles, and mammals were sold (86). The virus was detected on various surfaces, such as stall counters, sewage wells, and high-contact areas, indicating significant environmental contamination and the potential role of market conditions in viral transmission (87). While neither the putative animal reservoir for SARS-CoV-2 nor the possible role of an intermediate host in the emergence of COVID-19 has been determined, current evidence strongly suggests that the LAM in Wuhan played a critical role in the emergence of this pandemic pathogen (88, 89).

MERS-CoV is primarily transmitted through direct contact with infected camels or their secretions, including saliva, urine, or feces. A longitudinal study conducted in Abu Dhabi, United Arab Emirates, between 2014 and 2017 assessed the seroprevalence of MERS-CoV among various groups of LAM and slaughterhouse workers, including camel herders, camel salesmen, abattoir workers, and waste handlers. The study found seroprevalence rates ranging from 6% to 19% across three sampling periods, with camel salesmen exhibiting the highest seroprevalence (49%), followed by waste handlers (22%). These findings highlight the occupational risks associated with direct and prolonged contact with camels and their by-products, facilitating zoonotic transmission in TFM environments (90). A similar study in Qatar reported that 68% of 294 workers, primarily camel herders and market employees, were seropositive for MERS-CoV antibodies, further reinforcing the risk posed by these occupational exposures (91).

#### Other viruses

The sale and consumption of wild animal meat in TFMs have been identified as key drivers for virus transmission to humans (92–94). LAMs pose a particularly high risk, as viruses such as Ebola virus and *Henipaviruses* can be spread through direct contact with blood, saliva, and other bodily fluids from infected wild animals, as well as through consumption of contaminated food. Further research is needed to assess the presence and transmission dynamics of various zoonotic viruses in market settings. For instance, while studies on Lassa fever remain limited, one study in Nigeria examined the knowledge and behaviors of market traders in Lassa-endemic areas. The findings revealed that poor food handling practices—such as leaving food uncovered and accessible to rodents—could facilitate the spread of the virus through exposure to urine and feces from infected rodents in market environments (95).

### Vector-borne viruses

While vector-borne pathogens such as West Nile virus (WNV), Crimean-Congo hemorrhagic fever virus (CCHFV), Japanese encephalitis virus (JEV), Rift Valley fever virus, and Kyasanur Forest disease virus (KFDV) are rarely reported or studied in LAMs, their absence in surveillance data does not necessarily indicate a lack of risk. Indeed, LAMs create optimal conditions for the emergence and spread of vector-borne diseases, primarily through insect vectors such as mosquitoes and ticks. Mosquitoes from the *Aedes* and *Culex* genera thrive in urban environments where stagnant water pools often result from inadequate drainage and sanitation (96). These central conditions allow mosquito populations to proliferate, increasing the likelihood of viral transmission to humans. The close proximity of humans and animals in these markets further enhances the potential for mosquitoes to spread pathogens from infected animal hosts, such as bats and primates, to humans.

Ticks also play a critical role in zoonotic disease transmission, particularly for pathogens such as CCHFV and KFDV. Ticks often parasitize the livestock and wild animal species commonly sold in TFMs, which can serve as reservoirs for tick-borne diseases (97). Within the confined and often unhygienic conditions of these markets, ticks may have increased opportunities to attach to hosts, facilitating pathogen transmission (98).

A recent study conducted in Myanmar and Indonesia observed 16 mammalian species from seven families being traded in LAMs. In Myanmar, two-thirds of the animals were freshly slaughtered with improper storing of carcasses and low levels of cleaning, and the most common animal species included pigs (*Suidae*) and deer (*Cervidae*), both known reservoirs for arboviruses such as JEV and WNV. In Indonesia, squirrels (*Sciuridae*) and fruit bats (*Pteropodidae*) were the most frequently observed species, both of which have been linked to arboviruses such as JEV. The study also highlighted the presence of stagnant water and low levels of hygiene across the LAMs, which significantly contributed to mosquito proliferation. Principal component analysis revealed that CCHFV and WNV were strongly associated with markets in Myanmar, whereas JEV and Chikungunya were more prominent in LAMs in Indonesia (99).

### Bacterial threats in traditional food markets

Bacteria pathogens are also a critical concern in TFMs, where the sale of fresh meat, live animals, and prepared foods occurs in close proximity and often under conditions that lack adequate sanitation and food inspection. Bacteria, including *Salmonella* spp., *Escherichia coli*, *Brucella* spp., and *Leptospira* spp., are important when associated with TFMs, where they may contribute to the spread of foodborne disease and antimicrobial resistance (AMR). Although TFMs are not the primary sites where AMR develops, they can serve as important points of dissemination, especially in contexts where antibiotic use in food animals is poorly regulated and promotes the emergence and spread of resistant bacterial strains through contaminated products and market environments.

A study conducted in Lusaka and Kasumbalesa, Zambia, investigated the presence of zoonotic pathogens in small ruminants and assessed public health risks associated with informal markets. The researcher collected 237 blood samples from goats and sheep that were tested for antibodies to *Brucella* spp. and *Coxiella burnetii*, revealing seropositivity rates of 10.1% and 5.9%, respectively (100). These findings indicate active circulation of both pathogens in the animal source populations. Animals were commonly co-transported from various provinces and kept in close proximity at market pens, facilitating potential pathogen transmission. Slaughterhouse observations showed inadequate hygiene measures, such as the reuse of contaminated water for cleaning carcasses and lack of personal protective equipment among workers. Interviews with value chain actors highlighted limited awareness of *Brucella* spp. and *Coxiella burnetii* transmission risks and misconceptions about food safety, including the belief that boiling meat is sufficient to eliminate all hazards. These findings emphasize the importance of considering not just the wildlife trade but also the role of domestic animals in disease transmission and the need for comprehensive measures to mitigate zoonotic disease risks effectively.

In Bangladesh, a study revealed that *E. coli* was present in 64.4% of duck cloacal samples and in 100% of water samples from three LBMs. This indicates that ducks can be a source of *E. coli* contamination, which can spread throughout various areas of the market, including spaces where food is handled, posing a significant public health risk. These isolates demonstrated a high level of resistance to multiple antibiotics, including ampicillin, tetracycline, and nalidixic acid that likely originates during the animal production process on farms (101). Similarly, in Nigeria, *E. coli* was found in 80% of chicken samples from LBMs, with 56.3% of the isolates exhibiting multidrug resistance phenotypes (102). In Qatar, a 2018 study found *E. coli* in 90% of broiler chicken samples from LBMs and farms, with 15.5% of isolates resistant to colistin, an antibiotic of last resort (103).

TFMs may also provide an ideal environment for the transmission of *Leptospira* spp. due to poor waste management and inadequate pest control. Leptospirosis is commonly transmitted through water or soil contaminated with the urine of infected animals, including rodents, wildlife, and domestic animals. A study in Malaysia identified TFM workers as a high-risk group for leptospirosis, highlighting the role of environmental contamination in transmission of this pathogen (104).

*Salmonella* spp. contamination is also a major food safety concern in TFMs and is commonly associated with meat products and utensils. In Bangladesh, *Salmonella* was extensively studied in poultry processing environments across 29 LBMs. Researchers collected 870 samples, including carcass wash water, chopping board swabs (CBS), and knife swabs (KS). The prevalence of *Salmonella* was reported as 20% in carcass wash water, 19.3% in CBS, and 17.6% in KS. Notably, multidrug-resistant *Salmonella* strains were prevalent, with resistance rates of 72.4% in carcass wash water, 73.2% in CBS, and 68.6% in KS. The isolates were tested for eight virulence genes, and *S*. Enteritidis and untyped *Salmonella* strains were found to harbor all tested genes, while *S*. Typhimurium lacked two (sefA and spvC). Phenotypic resistance to antibiotics such as ciprofloxacin, tetracycline, and ampicillin was prominent (105, 106).

In Malaysia, researchers investigated the prevalence of *Salmonella* in poultry processing environments in TFMs. A total of 182 poultry and environmental samples were collected from TFMs and small-scale processing plants. The overall prevalence of *Salmonella* was 88.5%, with 100% contamination found in chicken carcasses, chicken cuts, and surfaces such as knives, chopping boards, display tables, and wash water. High contamination rates were also observed in defeathering machines (91.7%) and on aprons (66.7%). Seventeen serovars were identified, with *Salmonella* Albany (35.4%), *Salmonella* Corvallis (26.1%), and *Salmonella* Brancaster (22.9%) being the most common. The study highlighted biofilm formation as a key factor allowing *Salmonella* to persist for extended periods of time on processing surfaces, exacerbated by poor hygiene practices (107).

In Thailand, *Salmonella* was studied in chicken meat and environmental samples from three stalls at a major wet market. Researchers collected 130 samples, including chicken parts, food contact surfaces, and wastewater. Environmental contamination was linked to food preparation equipment such as cutting boards, knives, and working tables. Most isolates (*n* = 42) exhibited resistance to tetracycline, though none showed resistance to ceftriaxone. Multiple patterns of virulence genes were identified, indicating the presence of highly pathogenic strains (108).

*Campylobacter* spp., particularly *Campylobacter jejuni* and *Campylobacter coli*, are significant zoonotic pathogens frequently associated with foodborne illnesses. These bacteria are commonly found in the intestinal tracts of poultry and other animals, making them prevalent in TFMs where animal products are processed and sold. Studies in the Philippines and Bangladesh reported significant presence of *C. jejuni* and *C. coli* in TFMs. In Metropolitan Manila, *Campylobacter* was found in 118 chicken offal samples, with 13 sequence types identified, including the zoonotic ST-305 (35% prevalence). In Thailand, *Campylobacter* was detected in 61.5% of 296 chicken samples, with high rates of antimicrobial resistance identified, including 92.3% resistance to ciprofloxacin. Similarly, in Bangladesh, *Campylobacter* was present in 32% of samples from chick meconium, feed, drinking water, cloacal swab, farm broiler meat, floor swab, and market broiler meat, with 49% of *C. jejuni* isolates showing multidrug resistance (109–111).

### Parasites and protozoa in traditional food markets

While viruses and bacteria are widely recognized as significant public health threats stemming from LAMs, the contribution of parasites and protozoa to the zoonotic disease burden is understudied and likely underestimated. These pathogens are often regarded as “neglected” because they are almost absent from the global health agenda (112). However, they are also prevalent in various animal species sold in LAMs and pose significant risks to human health through direct contact, handling of contaminated products, or consumption of undercooked or raw meat. Numerous studies have demonstrated the widespread presence of zoonotic parasites in LAMs. For instance, a study conducted in Malaysia revealed a high prevalence of *Toxoplasma gondii* in ruminant meat sold in markets, with seropositivity rates of 54.7% in goats and 34.9% in sheep (113). In northern Thailand, *Echinostome metacercariae*—parasitic trematode larvae—were detected in 16.9% of *Viviparidae* snails sold in markets, presenting a potential risk for human infection through the consumption of raw or undercooked freshwater snails (114). Similarly, in Phnom Penh, Cambodia, *Gnathostoma spinigerum* advanced third-stage larvae were found in 60% of Chinese edible frogs sold in markets, highlighting the risks associated with amphibian consumption (115). Due to the wide variety of animals and animal-derived products, including fish, pork, beef, and poultry, sold in markets, testing should also be conducted for other parasites and protozoa with public health consequences, such as *Clonorchis sinensis, Opisthorchis* spp.*, Anisakis* spp.*, Taenia* spp.*, Fasciola hepatica, and Fasciola gigantica* (116–119).

## PATHOGEN MONITORING AND SURVEILLANCE IN TRADITIONAL FOOD MARKETS

Although there is not an extensive knowledge of all the zoonotic diseases that can be found in TFMs, it is important to highlight that there is still a lack of monitoring and surveillance in these settings worldwide. New technologies such as ES, which refers to the systematic monitoring of environmental samples to detect and analyze pathogens, have gained prominence as tools for public health, especially in tracking disease outbreaks and understanding pathogen dynamics in the environment. Its application in zoonotic disease surveillance has the potential to improve the detection and monitoring of known, unknown, and emerging infections of livestock and wildlife (120).

ES of surface waters using techniques like metagenomic sequencing theoretically allows for the tracking of specific strains of pathogens and the establishment of a link between disease cases and environmental samples without the need for cultivation (121). By integrating satellite-derived environmental variables with disease data, early warning systems have the potential to rapidly detect and predict disease outbreaks, enabling timely intervention and prevention (122).

Environmental sampling has been suggested as a more cost-effective and safer method of monitoring virus circulation in TFMs than direct animal sampling alone (123, 124). The inclusion of non-invasive sampling approaches coupled with portable sequencing systems (e.g., Oxford Nanopore’s MinION) and environmental sensors (e.g., air samplers, wastewater surveillance systems) can be integrated into continuous pathogen surveillance systems to enhance early detection, improve outbreak response times, and provide real-time data for monitoring zoonotic pathogens in high-risk environments such as TFMs. For instance, air and water sampling devices paired with portable sequencers can collect and identify genetic material in real time, providing actionable data for rapid and effective interventions. These technologies are less disruptive than traditional surveillance and can extend surveillance coverage in high-risk environments with frequent human-animal interactions, such as TFMs (125).

In numerous global settings, there is a growing trend of integrating routine COVID-19 surveillance programs with community-level environmental surveillance focused on detecting SARS-CoV-2 in wastewater samples. This approach mirrors similar efforts applied to other diseases and hazards like polio, typhoid, and antimicrobial resistance. The primary goal of environmental surveillance is to provide early alerts and supplementary insights regarding the prevalence of pathogens at high-risk human-animal interfaces. This encompasses identifying pathogen presence or absence, tracking trends in prevalence, and monitoring for emerging viral variants. The application of environmental surveillance can contribute to informed decision-making and facilitate the assessment of the impact of interventions designed to reduce risk (126).

## LIMITATIONS AND GAPS IN UNDERSTANDING ZOONOTIC PATHOGENS IN TRADITIONAL FOOD MARKETS

Despite their significance globally in food security, public health, and local economies, there is a considerable lack of data on the diversity of zoonotic pathogens in TFMs. While research efforts have been concentrated in Africa and Asia, other parts of the world remain largely absent from the scientific literature (69). This data gap limits our ability to fully understand pathogen dynamics in TFMs and address the associated challenges and risks. Critically, zoonotic disease risk from LAMs/TFMs is not limited to Africa and Asia, as exemplified by recent (i.e., 2025) AIV outbreaks in the USA, where authorities temporarily closed all live poultry markets in New York City and three suburban counties after detecting AIVs at seven markets. This demonstrates that risks associated with LAMs are not exclusive to low- or middle-income countries, and more work is needed to understand common drivers of risk in market environments (127).

The COVID-19 pandemic further complicated discourse around TFMs, reinforcing negative perceptions of these markets as potential hotspots for zoonotic disease emergence while simultaneously making surveillance and intervention efforts more difficult (128–131). A lack of cooperation among key stakeholders, often driven by distrust and concerns over cultural practices, has hindered progress in improving market safety and disease prevention.

In many countries, TFMs are not regarded as policy priorities, leading to limited research, oversight, and intervention efforts. Governments frequently allocate resources to other sectors, leaving these markets underregulated and poorly studied. Additionally, TFMs are often politically sensitive topics, deeply embedded in cultural traditions and local economies. Addressing the risks associated with these markets requires careful navigation to avoid social or political backlash.

One of the most significant barriers to improving TFM safety is the absence of clear legal and regulatory frameworks in many countries. Without well-defined policies, efforts to inspect, regulate, or enhance hygiene and food safety measures are inconsistent and often ineffective. This lack of oversight leaves TFMs operating in a regulatory gray area, where zoonotic disease prevention measures are minimal or entirely absent. Moreover, the diverse social, economic, and cultural factors associated with TFMs necessitate context-specific solutions. However, without comprehensive pathogen surveillance data, designing and implementing effective and tailored interventions will remain a challenge.

## CONCLUSION

TFMs serve as essential socio-economic and cultural hubs that provide affordable, fresh food and support the livelihoods of millions of people, globally. However, their significance is also accompanied by inherent risks, particularly those associated with the emergence and transmission of zoonotic diseases. The complexity of TFMs—characterized by close interactions between humans, live animals, and animal-derived products—creates conditions that can facilitate pathogen spillover. The presence of diverse wildlife species, live poultry, and other animals, combined with inadequate infrastructure and sanitation, further amplifies these risks. The role of TFMs in zoonotic disease emergence is evident in past outbreaks, with markets serving as key transmission nodes for pathogens with pandemic potential.

Efforts to mitigate risks in TFMs require a multi-faceted, One Health approach that integrates public health, animal health, and environmental health strategies. The implementation of robust surveillance systems, biosecurity measures, and hygiene protocols within TFMs is crucial for the early detection and control of zoonotic diseases. Improved market infrastructure and enhanced sanitation can significantly reduce pathogen transmission. Additionally, targeted education and training for vendors, market workers, and consumers can help promote safer food handling practices and reduce public health risks.

Despite these proposed interventions, significant challenges remain. Many TFMs operate within informal structures, lacking regulatory oversight, policy enforcement, and adequate investment in infrastructure. Moreover, data on the diversity and prevalence of zoonotic diseases in TFMs are limited, particularly in regions with high market activity but low surveillance capacity. Moving forward, a coordinated international effort is required to balance the economic and cultural importance of TFMs with public health and biosafety concerns. Strengthening global surveillance networks, fostering cross-sector collaboration, and incorporating innovative environmental monitoring technologies can enhance our ability to detect and respond to emerging threats. Future research should also prioritize underrepresented regions and pathogens to build a more comprehensive understanding of the risks associated with TFMs.

Ultimately, TFMs will continue to play a vital role in global food systems, but their sustainability and safety depend on integrating evidence-based strategies to mitigate zoonotic disease risks.

### Search strategy and selection criteria

We searched literature from major academic databases and gray sources to inform this narrative review. Our approach was based on expert knowledge rather than a systematic search protocol. We included peer-reviewed journal articles, books, reports, policy documents, and relevant studies on zoonotic disease risks in TFMs, particularly in low- and middle-income countries using search terms such as “traditional food markets,” “live animal markets,” “ zoonotic pathogens,” “pathogens,” “zoonotic diseases,” and “foodborne pathogens.” Sources published in English between 2000 and 2024 were considered. We did not restrict inclusion by study design and prioritized literature that provided descriptive data on pathogens, environmental contamination, transmission dynamics, or mitigation strategies within TFMs settings.
